# Exploratory association of OGTT area under the curve with early-night glycemic patterns and pharmacotherapy use in gestational diabetes mellitus

**DOI:** 10.3389/fendo.2026.1898511

**Published:** 2026-08-25

**Authors:** Jingxiang Feng, Shuyu Liu, Rongjie Lai, Zhaoyi Zhong, Yuzhi Chen, Weizhang Liang

**Affiliations:** Department of Obstetrics and Gynecology, Guangdong Provincial Key Laboratory of Major Obstetric Diseases, Guangdong Provincial Clinical Research Center for Obstetrics and Gynecology, Guangdong-Hong Kong-Macao Greater Bay Area Higher Education Joint Laboratory of Maternal-Fetal Medicine, The Third Afiiliated Hospital, Guangzhou Medical University, Guangzhou, China

**Keywords:** area under the curve, continuous glucose monitoring, gestational diabetes mellitus, glycemic variability, nocturnal glycemic patterns, oral glucose tolerance test, pharmacotherapy

## Abstract

**Background:**

In gestational diabetes mellitus (GDM), the relationship between baseline 75-g oral glucose tolerance test (OGTT) area under the curve (AUC), subsequent continuous glucose monitoring (CGM)-derived glycemic patterns, and pharmacotherapy use remains incompletely understood. This study aimed to examine the association of OGTT AUC with 14-day CGM profiles, with particular attention to nocturnal glycemic patterns, and pharmacotherapy use under routine clinical management.

**Methods:**

This single-center retrospective study included 64 singleton women with GDM identified from an institutional CGM registration cohort. Participants were stratified into quartiles according to baseline OGTT AUC. CGM-derived metrics were evaluated overall and across prespecified time windows. Hierarchical regression models were used to examine the associations of OGTT AUC with overall TAR and pharmacotherapy use. ROC analysis was performed as an exploratory assessment of discriminatory ability.

**Results:**

Women in the highest OGTT AUC quartile showed less favorable CGM-derived glycemic profiles, with higher hyperglycemic exposure and greater glycemic variability, particularly during waking hours (06:00-22:00) and the early-night (22:00-02:00) period. Higher OGTT AUC was associated with greater overall TAR in linear regression models and with higher odds of pharmacotherapy use in logistic regression models. The association persisted in sensitivity models additionally adjusted for CGM-derived mean glucose, although these models were interpreted cautiously because mean glucose may lie downstream of OGTT AUC. ROC analysis yielded an AUC of 0.770, with a potential cut-off of 17.34.

**Conclusions:**

In this exploratory CGM cohort of women with GDM, higher baseline OGTT AUC was associated with less favorable CGM-derived glycemic profiles, more apparent early-night glycemic abnormalities, and greater pharmacotherapy use under routine clinical management. OGTT AUC may help characterize subsequent glycemic burden and identify women who warrant closer glucose monitoring, but its clinical utility and candidate treatment-related thresholds require validation in larger prospective cohorts with standardized treatment-initiation criteria.

## 1 Introduction

Gestational diabetes mellitus (GDM) is defined as carbohydrate intolerance first diagnosed during pregnancy and has become a major health challenge worldwide. While the International Diabetes Federation (IDF) estimates that GDM occurs in approximately 14% of pregnancies globally (1), a comprehensive meta-analysis of Eastern and Southeastern Asia reported a comparable prevalence of 11.91% in China (2). Maternal hyperglycemia potentially causes adverse perinatal outcomes such as large-for-gestational-age (LGA) infants, preeclampsia, and preterm birth (3, 4). In the long term, GDM is closely associated with an increased risk of developing type 2 diabetes mellitus (T2DM), metabolic syndrome, and cardiovascular disease in affected mothers (5). Furthermore, it predisposes offspring to childhood obesity and glucose intolerance (6). To mitigate these risks, timely diagnosis and stringent glycemic management are imperative. The 75-gram oral glucose tolerance test (75-g OGTT) remains the gold standard for GDM diagnosis, yet it faces pitfalls including poor reproducibility and a reliance on fixed static thresholds (7). Driven by high intra-individual variation, these static measurements frequently mask individualized glycemic excursions and fail to account for the heterogeneous metabolic responses to habitual dietary intake and daily physical activity (7). Notably, glycemic elevations at different OGTT intervals reflect distinct pathophysiological mechanisms, such as varying degrees of impaired insulin secretion versus insulin resistance (8). To account for these diverse metabolic profiles while mitigating the instability of single-point measurements, we utilized the area under the curve (AUC) as a holistic metric. This approach integrates the entire 2-hour profile, providing a more stable and comprehensive representation of the total glycemic burden than discrete static thresholds.

Previous studies have suggested that OGTT AUC may provide a more integrated measure of post-load glycemic exposure than isolated glucose thresholds. Specifically, a study in China demonstrated that an elevated AUC is positively correlated with an increased risk of several neonatal outcomes, suggesting its utility in unmasking undetected hyperglycemia across the entire pregnant population (9). In parallel, other findings indicate that higher OGTT AUC has been associated with composite adverse pregnancy outcomes, reflecting the severity of maternal glucose intolerance more accurately than the mere count of abnormal values during the diagnostic test (10).

Nocturnal glucose dynamics are essential to comprehensive glycemic assessment, yet they receive relatively little clinical attention, largely due to the inherent limitations of traditional self-monitoring of blood glucose (SMBG). A study in Spain has shown that nocturnal hyperglycemia accounts for only a small fraction of the total monitoring duration (11). However, Law and colleagues (12) demonstrated that suboptimal nocturnal glucose remains a potent predictor of LGA infants, even among women achieving daytime glycemic targets. Nevertheless, whether baseline OGTT AUC is associated with subsequent CGM-derived nocturnal glucose patterns, particularly during distinct nighttime subperiods, remains unclear.

Therefore, this study aimed to explore the association between baseline OGTT AUC and CGM-derived glycemic patterns in women with GDM, with particular attention to prespecified nocturnal time windows. We also examined whether OGTT AUC was associated with pharmacotherapy use and maternal-neonatal outcomes under routine clinical management.

## 2 Methods

### 2.1 Participants and study design

This was a single-center, retrospective observational study conducted at the Department of Obstetrics and Gynecology of The Third Afiiliated Hospital of Guangzhou Medical University. Our hospital provides coverage to more than 10 million inhabitants, with an average of approximately 8,500 deliveries per year.

**Figure 1:**
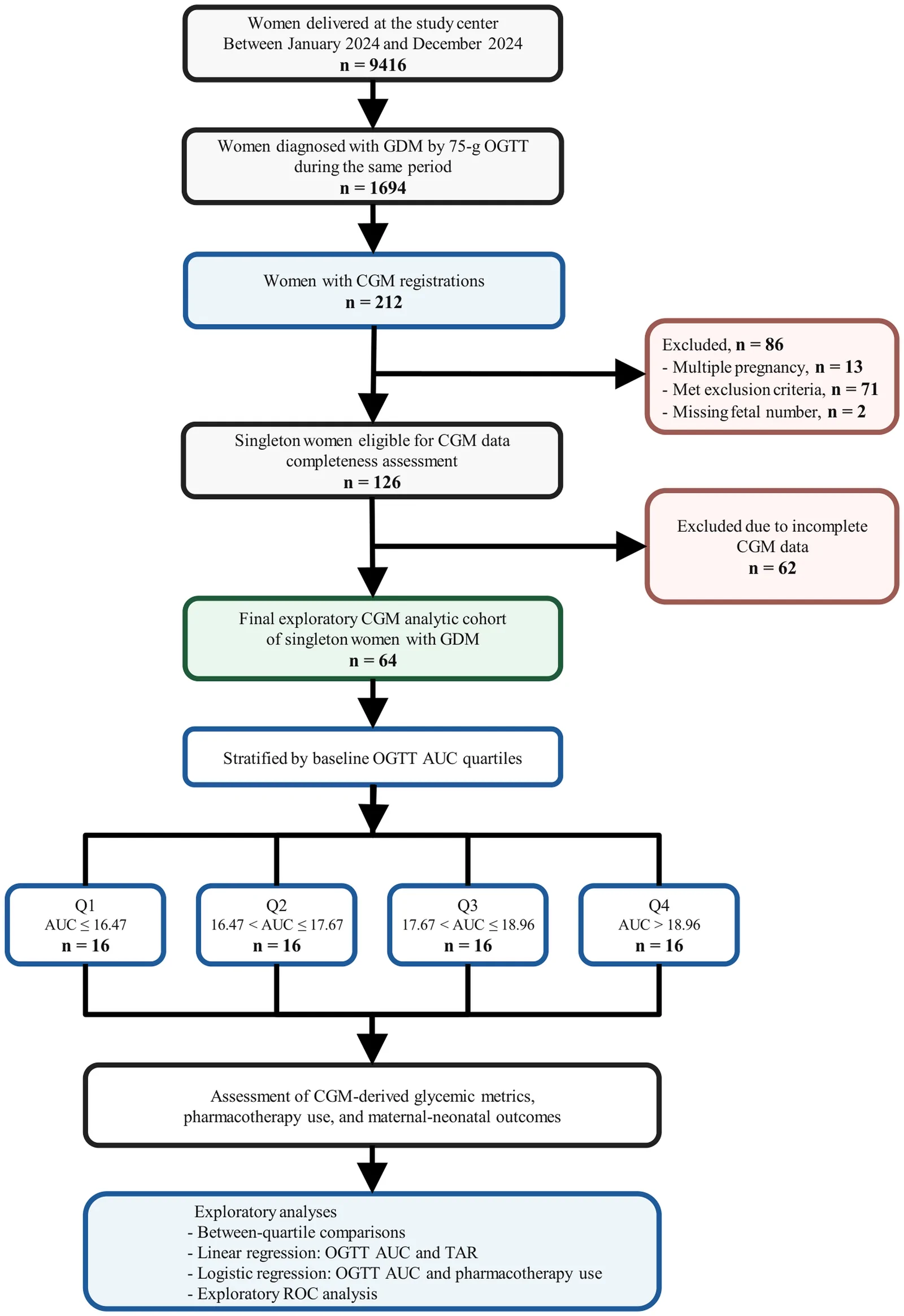
Flowchart of participant identification and CGM analytic cohort construction. The number of women diagnosed with GDM during the study period is shown to provide source-population context. The final analytic cohort was constructed from women with CGM registrations after applying eligibility criteria and CGM data completeness assessment. Participants were stratified into quartiles according to baseline OGTT AUC. GDM, gestational diabetes mellitus; OGTT, oral glucose tolerance test; CGM, continuous glucose monitoring; AUC, area under the curve; TAR, time above range; ROC, receiver operating characteristic.

We retrospectively constructed a CGM analytic cohort from women with CGM registrations at our center between January 2024 and December 2024. During the same period, 9,416 women delivered at the study center, and 1,694 women were diagnosed with GDM by 75-g OGTT, providing the source-population context. GDM was diagnosed according to the World Health Organization (WHO) 2013 criteria (13) using a standard 75-g OGTT after an overnight fast. Diagnosis was confirmed if at least one of the following venous plasma glucose thresholds was met: fasting ≥ 5.1 mmol/L, 1-hour ≥ 10.0 mmol/L, or 2-hour ≥ 8.5 mmol/L. Inclusion criteria were: (1) Singleton pregnancy; (2) Age ≥ 18 years; (3) availability of CGM data sufficient for 14-day profile assessment within 1 week of diagnosis. The minimum acceptable monitoring duration was 14 complete monitoring days. Exclusion criteria were: (1) pre-existing diabetes; (2) chronic hypertension; (3) use of glucocorticoids; and (4) missing fetal number information precluding confirmation of singleton pregnancy. Participants with incomplete CGM data were excluded because sensor-related problems led to interruption of CGM monitoring and insufficient monitoring duration. A total of 64 pregnant women diagnosed with GDM between 24 and 28 weeks of gestation were included in the CGM analytic cohort. The participant identification and analytic cohort construction process is shown in **Figure 1**.

This study was approved by the Ethics Committee of The Third Affiliated Hospital of Guangzhou Medical University (Approval No. YL-20260522000451). Due to the retrospective observational nature of the study and the use of de-identified clinical data, the requirement for written informed consent was waived by the Ethics Committee. The study was conducted in strict accordance with the Declaration of Helsinki.

### 2.2 Clinical data collection

Baseline clinical characteristics were retrieved from the hospital’s electronic medical record system. Collected data included maternal age, pre-gestational body mass index (pBMI), gravidity, and parity, OGTT glucose values, pharmacotherapy use, and maternal-neonatal outcomes. OGTT diagnosis dates were obtained from laboratory records and the electronic medical record system. Maternal pBMI was calculated as pre-pregnancy weight divided by the square of height. These maternal characteristics were used to describe the study population and served as baseline covariates in exploratory regression analyses.

### 2.3 OGTT AUC calculation and CGM monitoring

Participants underwent a 75-g OGTT during gestational weeks 24-28 after overnight fasting for at least 8 h. Blood glucose values were measured at 0, 1, and 2 h. The AUC of the time-blood glucose curve of the OGTT was measured using the trapezoidal rule as follows: AUC = ((0 h blood glucose + 1 h blood glucose)/2 + (1 h blood glucose + 2 h blood glucose)/2). Participants were stratified into quartiles according to baseline OGTT AUC values: Q1 (AUC ≤ 16.47), Q2 (16.47 < AUC ≤ 17.67), Q3 (17.67 < AUC ≤ 18.96), and Q4 (AUC > 18.96). These quartiles represented relative rankings within the present cohort rather than externally validated clinical categories.

Within 1 week after OGTT diagnosis, a SiBio continuous glucose monitoring (CGM) system (GS1; Shenzhen Sibionics Technology Co., Ltd., Shenzhen, China) was applied for clinical glucose monitoring. Sensors were placed on the back of the upper arm, and glucose data were recorded over a 14-day monitoring period. CGM start and end dates were obtained from CGM registration or export records. Interstitial glucose levels were captured at 5-minute intervals and automatically converted into blood glucose equivalents. Across the complete 14-day records included in the analysis, 82 isolated 5-minute CGM readings were missing, corresponding to 0.032% of the expected readings; available valid readings were used without imputation. During this period, participants maintained their routine diet and physical activity in accordance with standard clinical guidance. To evaluate both overall and period-specific glycemic patterns, CGM data were analyzed over the monitoring period and initially segmented into the waking hours (06:00-22:00) and night-time (22:00-06:00) periods. The nighttime period was then further subdivided into the early-night phase (22:00-02:00) and the late-night phase (02:00-06:00). For each participant, glycemic metrics included mean glucose (MG), standard deviation (SD), time above range (TAR; blood glucose > 7.8 mmol/L), time in range (TIR; blood glucose ≥ 3.9 and ≤ 7.8 mmol/L), and the standard deviation of daily TAR and TIR (SD_TAR_ and SD_TIR_). Furthermore, the mean amplitude of glycemic excursions (MAGE) was calculated exclusively for the overall monitoring period, as shorter segments could preclude the reliable capture of complete glycemic excursions.

### 2.4 AGP construction

To visualize continuous glycemic patterns, 24-hour ambulatory glucose profiles (AGPs) were constructed. Raw 5-minute CGM readings were aligned to the nearest 5-minute clock bin, yielding 288 time bins per day. A two-stage aggregation approach was used: first, each participant’s mean glucose value was calculated for each 5-minute clock bin across available monitoring days included in the analysis; second, group-level means and SDs were calculated for each OGTT AUC quartile. Shaded SD bands were displayed only for the extreme quartiles (Q1 and Q4) to illustrate variability without visual clutter.

### 2.5 Pharmacotherapy criteria

After OGTT diagnosis, all participants received standardized dietary counseling and lifestyle intervention as part of routine clinical care. Pharmacotherapy use was ascertained from electronic medical records and prescription records; treatment-initiation dates were recorded when reliably documented. Pharmacotherapy, including insulin or metformin, was initiated by the attending physician when glycemic targets were not achieved despite lifestyle intervention, in accordance with local clinical guidelines and based primarily on self-monitored capillary blood glucose profiles and clinical assessment. The criteria included:

1. Fasting capillary blood glucose ≥ 5.3 mmol/L on two separate occasions;
2. 2-hour postprandial capillary blood glucose ≥ 6.7 mmol/L on two separate occasions;
3. Persistent nocturnal hyperglycemia (> 7.0 mmol/L) detected by self-monitored blood glucose (SMBG).

Treatment decisions were not based on the OGTT AUC calculated for this study. Because of the retrospective design and incomplete documentation of treatment-initiation dates, the exact temporal sequence between CGM monitoring and pharmacotherapy initiation could not be reliably verified for all participants.

### 2.6 Maternal and neonatal outcomes

Following the completion of pregnancy, clinical data regarding GDM management and perinatal outcomes were systematically retrieved from medical records. Maternal complications recorded included pregnancy-induced hypertension (systolic blood pressure ≥ 140 mmHg or diastolic blood pressure ≥ 90 mmHg after 20 weeks of gestation in previously normotensive women), preeclampsia (pregnancy-induced hypertension accompanied by proteinuria ≥ 300 mg/24 h or ≥ 30 mg/mmol creatinine), polyhydramnios (amniotic fluid index ≥ 25 cm or maximum vertical pocket ≥ 8 cm), and postpartum hemorrhage (estimated blood loss ≥ 500 mL following vaginal delivery or ≥ 1000 mL following cesarean section within 24 hours). Mode of delivery was categorized as either vaginal delivery or cesarean section. Neonatal outcomes and associated complications encompassed preterm birth (delivery between 28 and 36 + 6 weeks of gestation), miscarriage, stillbirth, and macrosomia (birth weight ≥ 4000 g).

### 2.7 Statistical analysis

Statistical analyses were performed using IBM SPSS Statistics version 25 (IBM Corp., Armonk, NY, USA). Graphical illustrations and ambulatory glucose profile renderings were generated using Python (version 3.12.0; Python Software Foundation) with the pandas (version 2.3.3) and matplotlib (version 3.11.0) libraries. Continuous variables were presented as mean ± SD or median (interquartile range) according to their distribution. For group comparisons, participants were stratified into quartiles (Q1-Q4) based on their baseline OGTT AUC values. For continuous variables, one-way ANOVA was used when the data were approximately normally distributed and the homogeneity of variance assumption was satisfied; otherwise, the Kruskal-Wallis test was used. For categorical variables, the chi-square test was used when all expected cell counts were ≥ 5; otherwise, Fisher’s exact test was applied. When the overall test was statistically significant, Tukey’s honestly significant difference test was used for *post-hoc* pairwise comparisons after one-way ANOVA, whereas Dunn’s *post-hoc* test with Bonferroni adjustment was used after Kruskal-Wallis tests. For categorical variables with significant overall chi-square tests, *post-hoc* pairwise comparisons of column proportions were performed in SPSS with Bonferroni-adjusted P values. Superscript letters in **Tables 1**–**3** indicate statistically significant *post-hoc* pairwise comparisons after the corresponding adjustment procedures. Regression analyses were performed to evaluate the associations of OGTT AUC with overall TAR and pharmacotherapy use. For the linear regression models with overall TAR as the dependent variable, Model 1 was unadjusted, Model 2 was adjusted for maternal age and pre-pregnancy BMI, and Model 3 additionally included CGM-derived mean glucose as a sensitivity analysis. For the logistic regression models with pharmacotherapy use as the dependent variable, the same hierarchical modeling strategy was applied. Regression results are reported as unstandardized coefficients (*B*) for TAR models and odds ratios (ORs) for pharmacotherapy models, with 95% confidence intervals (CIs) and *P* values. Adjusted R² was reported for linear regression models to describe model explanatory ability. Collinearity was assessed using variance inflation factors (VIFs). ROC curve analysis was performed as an assessment of the discriminatory ability of OGTT AUC for pharmacotherapy use. The cut-off value was identified using the Youden index and was considered a candidate, sample-dependent threshold. Given the exploratory nature of this study and the number of CGM metrics and time windows examined, no additional multiplicity correction was applied across different CGM metrics or predefined time windows; therefore, period-specific findings were considered hypothesis-generating.

**Table 1:** Maternal characteristics stratified by OGTT AUC quartiles.

Characteristic	Total (n = 64)	Q1 (n = 16)	Q2 (n = 16)	Q3 (n = 16)	Q4 (n = 16)	*P* value
Age (years)	34.50 (31.00-38.00)	36.00 (33.00-37.00)	34.50 (31.00-38.00)	33.50 (30.00-38.75)	34.00 (32.25-40.50)	0.895
BMI (kg/m2)	22.78 ± 2.65	22.41 ± 2.18	22.26 ± 3.26	23.74 ± 2.39	22.72 ± 2.64	0.395
Gravidity	2.00 (1.00-3.00)	2.00 (1.00-3.00)	2.00 (1.00-3.00)	2.00 (1.00-3.75)	2.50 (1.25-3.00)	0.904
Parity	1.00 (1.00-2.00)	1.50 (1.00-2.00)	1.00 (1.00-2.00)	1.00 (1.00-2.00)	1.00 (1.00-2.00)	0.912
75-g OGTT						
(mmol/L)						
FPG	4.67 (4.40-5.37)	4.42 (4.13-4.67)	4.52 (4.36-4.76)	5.04 (4.81-5.39) ^a^	5.45 (4.37-6.37) ^a^	0.001
PG-1h	10.67 (9.89-11.55)	9.24 (8.52-10.05)	10.32 (9.99-10.62)	11.13 (10.78-11.42) ^a^	12.88 (11.99-13.60) ^a,b,c^	< 0.001
PG-2h	9.32 (8.63-10.98)	8.68 (7.08-9.31)	9.09 (8.50-9.28)	9.63 (8.38-10.17)	12.36 (11.10-13.28) ^a,b,c^	< 0.001

Continuous variables are presented as mean ± SD or median (interquartile range) according to their distribution. Superscript letters indicate significant Bonferroni-adjusted *post-hoc* pairwise comparisons: ^a^P < 0.05 vs Q1; ^b^P < 0.05 vs Q2; ^c^P < 0.05 vs Q3.

**Table 2:** CGM metrics stratified by OGTT AUC quartiles.

Characteristic	Q1	Q2	Q3	Q4	*P* value
Over 14 days					
MAGE (mmol/L)	2.12 ± 0.47	2.35 ± 0.47	1.98 ± 0.29	2.79 ± 0.75 a^,c^	0.002
MG (mmol/L)	5.63 ± 0.32	5.71 ± 0.42	5.64 ± 0.25	5.94 ± 0.28 a	0.035
SD (mmol/L)	1.02 ± 0.21	1.11 ± 0.29	0.95 ± 0.15	1.27 ± 0.27c	0.009
TAR (%)	4.03 (0.73-5.12)	4.58 (0.80-8.25)	1.54 (0.92-3.48)	7.71 (4.30-12.53) ^a,c^	0.004
SD_TAR_ (%)	3.19 (1.21-5.61)	3.48 (1.24-6.90)	2.46 (1.66-3.92)	6.04 (4.25-8.62) ^c^	0.011
TIR (%)	94.64 ± 3.62	92.02 ± 6.41	95.97 ± 2.83	89.68 ± 5.91c	0.006
SD_TIR_ (%)	5.55 (3.56-9.18)	4.99 (3.56-8.29)	3.91 (3.10-6.32)	6.87 (5.19-10.18)	0.137
Time segmented					
Waking hours					
MG (mmol/L)	5.79 ± 0.33	5.84 ± 0.39	5.73 ± 0.22	6.08 ± 0.37 c	0.021
SD (mmol/L)	1.05 ± 0.21	1.15 ± 0.29	0.98 ± 0.13	1.32 ± 0.31 c	0.006
TAR (%)	5.26 (1.09-7.26)	5.71 (1.18-11.59)	2.23 (1.29-4.60)	10.54 (4.72-14.96) ^c^	0.003
SD_TAR_ (%)	4.77 (1.81-8.28)	4.93 (1.84-8.84)	3.22 (2.50-5.70)	7.57 (5.31-10.49) ^c^	0.035
TIR (%)	93.90 ± 4.13	91.57 ± 6.49	95.66 ± 2.98	87.38 ± 7.41 a, c	0.003
SD_TIR_ (%)	6.72 (3.97-10.77)	5.83 (3.25-9.22)	5.49 (3.38-8.25)	7.75 (6.77-10.45)	0.190
Nighttime period					
MG (mmol/L)	5.32 (5.11-5.57)	5.25 (5.09-5.45)	5.48 (5.14-5.69)	5.61 (5.35-5.94)	0.032
SD (mmol/L)	0.80 ± 0.26	0.89 ± 0.31	0.82 ± 0.22	1.03 ± 0.22	0.047
TAR (%)	0.71 (0.00-2.01)	1.27 (0.00-5.04)	0.30 (0.02-1.67)	3.20 (1.90-7.05)	0.033
SD_TAR_ (%)	1.73 (0.00-3.88)	2.97 (0.00-7.57)	1.11 (0.07-4.42)	6.28 (5.09-10.10) ^a^	0.022
TIR (%)	97.36 (95.20-98.05)	95.83 (88.53-98.88)	98.03 (96.16-98.36)	94.94 (90.83-97.52)	0.299
SD_TIR_ (%)	6.93 (3.84-9.57)	6.70 (3.39-14.99)	4.99 (3.70-7.89)	9.69 (5.11-11.58)	0.494
Early-night period					
MG (mmol/L)	5.67 (5.33-5.98)	5.56 (5.30-5.88)	5.76 (5.51-6.18)	6.16 (5.92-6.27) a, b	0.015
SD (mmol/L)	0.80 ± 0.25	0.90 ± 0.30	0.83 ± 0.20	1.03 ± 0.24 a	0.049
TAR (%)	1.42 (0.00-2.61)	2.31 (0.00-4.28)	0.55 (0.04-3.35)	6.10 (2.23-12.02) ^a^	0.034
SD_TAR_ (%)	3.45 (0.00-7.24)	5.95 (0.00-10.84)	1.99 (0.14-8.85)	12.13 (6.27-17.37) ^a^	0.030
TIR (%)	97.17 (95.02-98.03)	96.28 (89.40-99.19)	97.85 (94.77-99.22)	92.71 (87.20-95.05)	0.143
SD_TIR_ (%)	7.09 (4.57-12.42)	7.16 (2.29-15.46)	5.58 (2.29-14.63)	12.64 (10.13-17.34)	0.285
Late-night period					
MG (mmol/L)	4.88 (4.71-5.29)	4.97 (4.83-5.11)	4.94 (4.74-5.31)	5.20 (4.96-5.32)	0.398
SD (mmol/L)	0.51 (0.38-0.86)	0.61 (0.46-0.81)	0.53 (0.42-0.65)	0.70 (0.58-0.83)	0.127
TAR (%)	0.00 (0.00-0.26)	0.00 (0.00-0.78)	0.00 (0.00-0.00)	0.00 (0.00-1.71)	0.479
SD_TAR_ (%)	0.00 (0.00-0.97)	0.00 (0.00-2.92)	0.00 (0.00-0.00)	0.00 (0.00-6.40)	0.460
TIR (%)	97.10 (94.98-99.15)	95.09 (91.74-99.19)	97.92 (96.77-99.33)	97.10 (95.46-98.96)	0.370
SD_TIR_ (%)	6.72 (2.57-10.99)	11.03 (2.34-19.95)	6.53 (2.48-9.16)	7.08 (2.74-14.97)	0.560

^a^*P* < 0.05 compared with Q1; ^b^*P* < 0.05 compared with Q2; ^c^*P* < 0.05 compared with Q3.

**Table 3:** Pregnancy and perinatal outcomes stratified by OGTT AUC quartiles.

Outcomes	Q1 (n = 16)	Q2 (n = 16)	Q3 (n = 16)	Q4 (n = 16)	*P* value
Pharmacological treatment, n (%)	3 (18.8)	3 (18.8)	8 (50.0)	11 (68.8) a, b	0.006
Cesarean section, n (%)	10 (62.5)	5 (31.3)	5 (31.3)	9 (56.3)	0.155
Pregnancy-induced hypertension, n (%)	0 (0.0)	1 (6.3)	2 (12.5)	2 (12.5)	0.742
Postpartum hemorrhage, n (%)	1 (6.3)	2 (12.5)	1 (6.3)	1 (6.3)	1.000
Polyhydramnios, n (%)	2 (12.5)	1 (6.3)	0 (0.0)	1 (6.3)	0.897
Macrosomia, n (%)	1 (6.3)	1 (6.3)	2 (12.5)	2 (12.5)	1.000
Preterm birth, n (%)	1 (6.3)	1 (6.3)	0 (0.0)	0 (0.0)	1.000

Data are presented as n (%). Superscript letters indicate significant Bonferroni-adjusted pairwise comparisons of column proportions: ^a^P < 0.05 versus Q1; ^b^P < 0.05 versus Q2.

## 3 Results

### 3.1 Baseline characteristics and patient grouping

The final CGM analytic cohort included 64 women diagnosed with GDM by 75-g OGTT between 24 and 28 weeks of gestation. Of the 64 patients, the median area under the 75-g OGTT curve was 17.67 (range: 13.88-41.70). Based on the prespecified quartile grouping, the cutoff values for the four groups (n = 16 per group) were: Q1 (≤ 16.47), Q2 (> 16.47 to ≤ 17.67), Q3 (> 17.67 to ≤ 18.96), and Q4 (> 18.96).

Baseline maternal characteristics, including maternal age, pre-pregnancy BMI, gravidity, and parity, did not differ significantly across OGTT AUC quartiles (all P > 0.05; **Table 1**). As expected, OGTT glucose values differed across AUC quartiles, with higher values generally observed in the higher AUC groups (**Table 1**).

### 3.2 Glucose monitoring

#### 3.2.1 14-day duration glycemic profiles

Over the 14-day continuous glucose monitoring period, significant inter-group differences were observed in both hyperglycemia exposure and overall glycemic variability (**Table 2**). *Post-hoc* pairwise comparisons indicated that the Q4 group exhibited a higher mean glucose (MG) compared with the Q1 group. Furthermore, the Q4 group demonstrated significantly increased time above range (TAR) and mean amplitude of glycemic excursions (MAGE) relative to both the Q1 and Q3 groups, alongside significantly higher SD and SD_TAR_, but lower TIR, than those observed in the Q3 group (all *P* < 0.05).

No significant differences were found in the remaining pairwise comparisons. Because most significant pairwise differences involved Q4, these findings should be interpreted as exploratory evidence that less favorable CGM-derived glycemic profiles were concentrated among women in the highest OGTT AUC quartile (Q4).

#### 3.2.2 Time-segmented glycemic patterns

**Figure 2:**
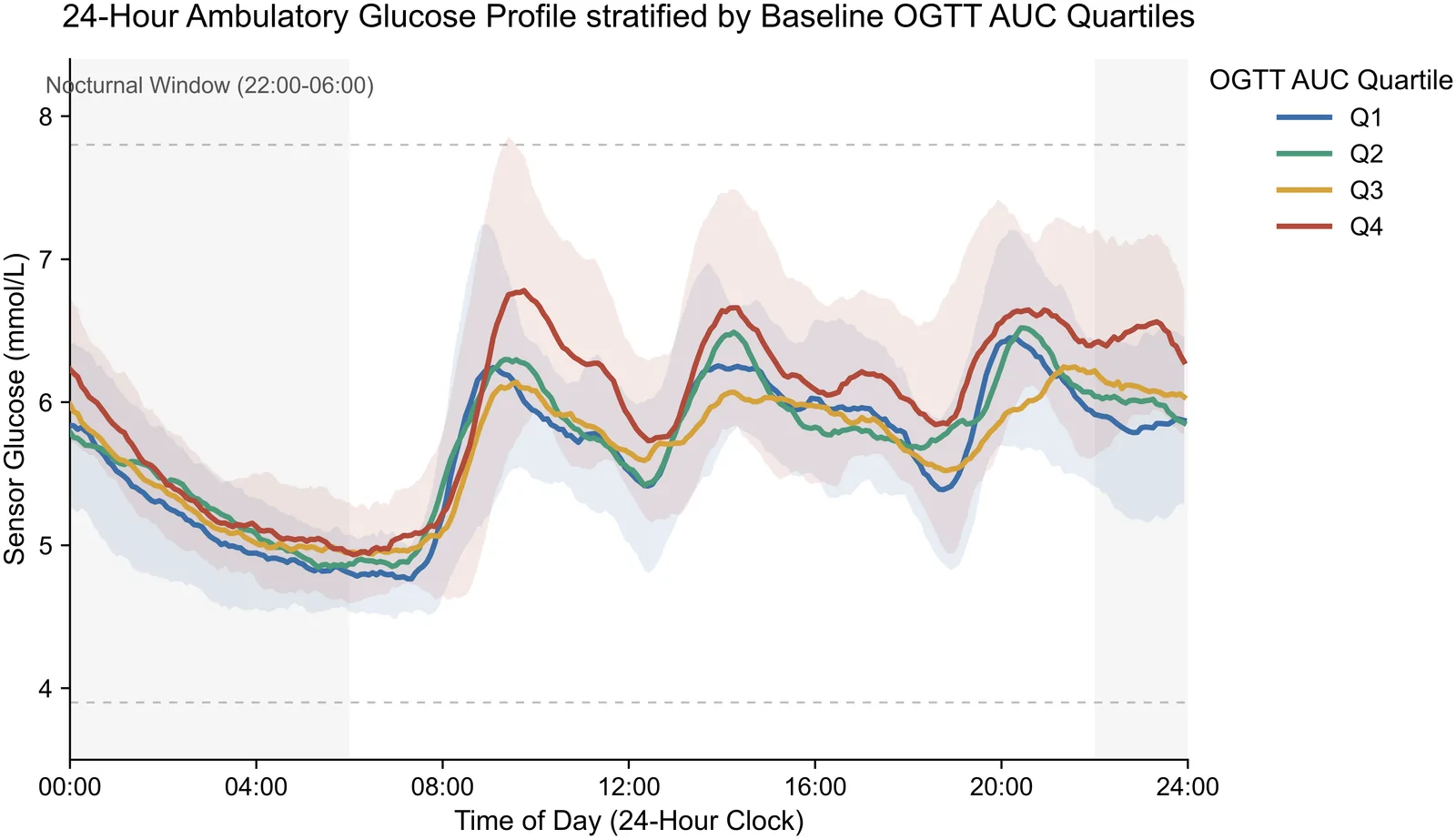
24-hour ambulatory glucose profile (AGP) stratified by baseline OGTT AUC quartiles. Continuous curves represent the group-level mean sensor glucose values captured at 5-minute intervals (288 points/day) across the monitoring period (n = 64). Shaded ribbons represent the standard deviation (SD) bands, displayed exclusively for the extreme groups (Q1, light blue; Q4, light red) to contrast glycemic variability without visual clutter. The shaded vertical regions highlight the designated Nocturnal Window (22:00-06:00). Horizontal dashed gray lines indicate clinical reference thresholds at 3.9 mmol/L and 7.8 mmol/L, respectively. AUC, area under the curve; CGM, continuous glucose monitoring; OGTT, oral glucose tolerance test; SD, standard deviation.

The 24-hour AGP visually illustrated distinct temporal glucose patterns across OGTT AUC quartiles, with the Q4 group showing higher mean glucose levels particularly during waking hours and the early-night period (**Figure 2**). During the waking hours (06:00-22:00), significant inter-group differences were observed in multiple glycemic parameters. *Post-hoc* comparisons revealed that compared with the Q3 group, the Q4 group exhibited elevated MG, SD, TAR, and SD_TAR_ (all *P* < 0.05). In contrast, the TIR in the Q4 group was significantly lower relative to the Q1 and Q3 groups (all *P* < 0.05).

While global trends of glycemic abnormalities extended into the entire nighttime period (22:00-06:00), *post-hoc* pairwise comparisons failed to identify statistically significant differences for most metrics between specific groups; the only exception was that the Q4 group exhibited a higher SD_TAR_ relative to the Q1 group (*P* < 0.05). However, when the nighttime was further stratified, a more apparent temporal pattern was observed. During the early night (22:00-02:00), the MG in the Q4 group was significantly higher than that in the Q1 and Q2 groups. Furthermore, the Q4 group demonstrated significantly greater SD, TAR, and SD_TAR_ compared to the Q1 group (all P < 0.05). Notably, these between-group differences were no longer evident during the late night (02:00-06:00), with no significant differences observed. In addition, TIR and SD_TIR_ showed no significant between-group differences during the overall nighttime, early-night, or late-night windows (all P > 0.05). These temporal trends suggest that early-night differences may be more apparent among women with higher baseline OGTT AUC, warranting further investigation.

### 3.3 Pregnancy progress, delivery, and maternal-fetal complications

During pregnancy, 25 participants (39.1%) received pharmacological treatment with insulin or metformin. Pregnancy-induced hypertension occurred in 5 participants (7.8%). Postpartum hemorrhage was observed in 5 patients (7.8%). There were 4 cases (6.3%) of polyhydramnios, and none developed preeclampsia. Cesarean section was performed in 29 patients (45.3%), and vaginal delivery occurred in 35 patients (54.7%).

Regarding perinatal outcomes, the mean newborn weight was 3305.23 ± 417.14 g, the incidence of macrosomia was 6 (9.4%), and that of preterm birth was 2 (3.1%). Notably, no other major adverse perinatal outcomes, including miscarriage, intrauterine fetal demise, or stillbirth, were recorded.

No significant differences were observed when comparing adverse perinatal outcomes among the four glycemic groups (all *P* > 0.05; **Table 3**). However, pharmacotherapy use differed significantly across the groups, with the Q4 group exhibiting a higher rate compared to the Q1 and Q2 groups (68.8% vs. 18.8% and 18.8%, respectively; *P* = 0.006).

### 3.4 Exploratory regression and ROC analyses

**Figure 3:**
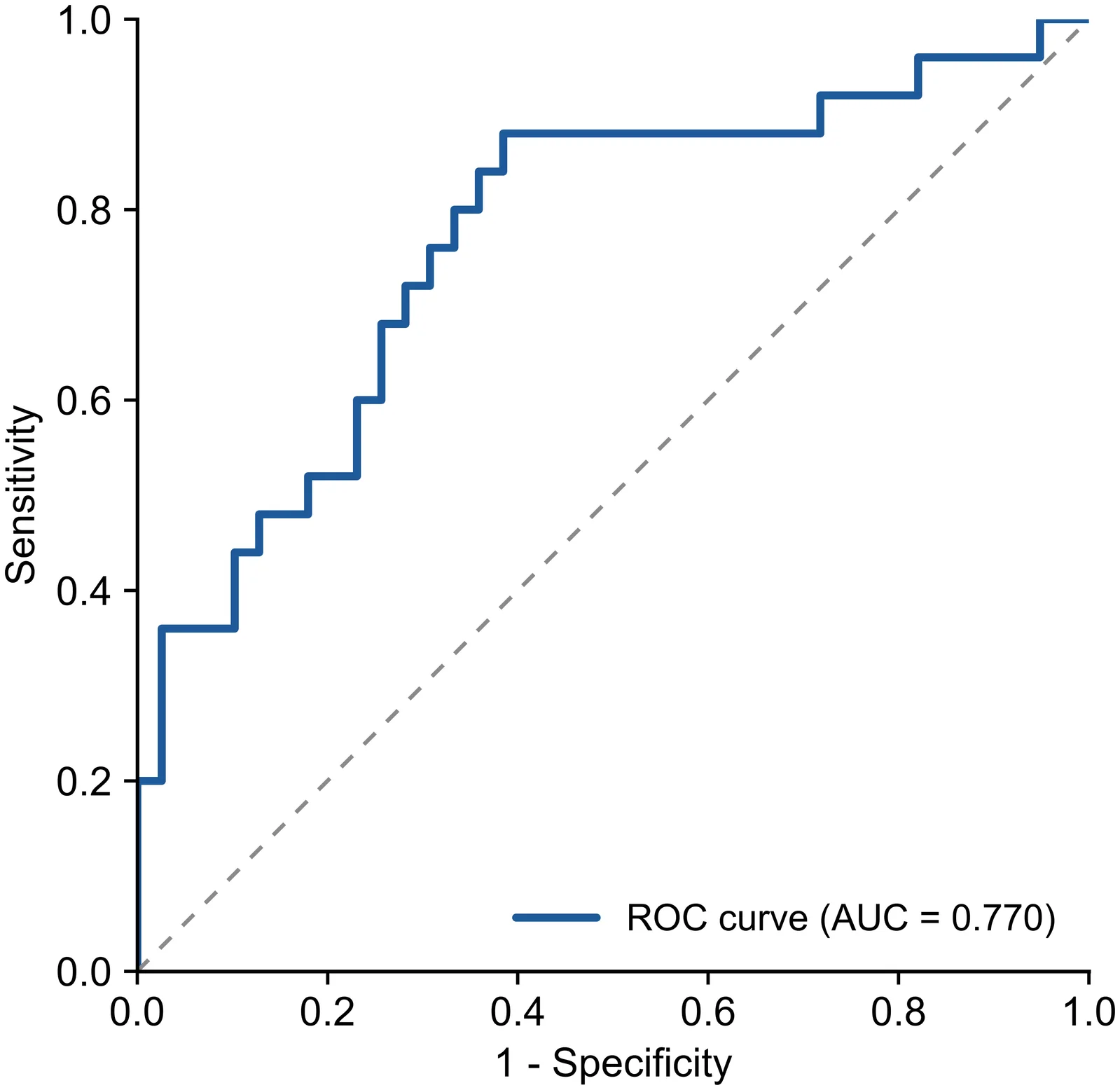
Exploratory receiver operating characteristic (ROC) curve analysis. The solid blue line represents the ROC curve evaluating the discriminatory ability of baseline OGTT AUC for pharmacotherapy use during pregnancy. The dashed grey diagonal line indicates the reference line for random chance. The overall area under the curve (AUC) is 0.770 (95% CI: 0.648-0.892). Based on this analysis, a candidate, sample-dependent cut-off value of 17.34 was identified, yielding a sensitivity of 88.0% and a specificity of 61.5% in this preliminary cohort. AUC, area under the curve; CI, confidence interval.

In linear regression analyses, higher OGTT AUC was consistently associated with higher overall TAR (**Table 4**). This association was observed in the unadjusted model (*B* = 0.551, 95% CI: 0.234-0.867, *P* = 0.001) and remained essentially unchanged after adjustment for maternal age and pre-pregnancy BMI (*B* = 0.555, 95% CI: 0.239-0.871, *P* = 0.001). In the sensitivity model additionally adjusted for CGM-derived mean glucose, the overall model showed the highest explanatory ability (adjusted R² = 0.738), while the magnitude of the association for OGTT AUC was attenuated but remained statistically significant (*B* = 0.196, 95% CI: 0.009-0.383, *P* = 0.040). This suggests that mean glucose explained a substantial proportion of the variation in overall TAR, but the association between OGTT AUC and TAR was not entirely explained by average CGM glucose levels. Similarly, higher OGTT AUC was associated with greater pharmacotherapy use. The association was significant in the unadjusted model (OR = 1.587, 95% CI: 1.187-2.121, *P* = 0.002). In the minimally adjusted model, each 1-unit higher 75-g OGTT AUC was associated with 58.5% higher odds of pharmacotherapy use after adjustment for maternal age and pre-pregnancy BMI (OR = 1.585, 95% CI: 1.185-2.120, *P* = 0.002). After further inclusion of CGM-derived mean glucose in Model 3, OGTT AUC remained associated with pharmacotherapy use (OR = 1.507, 95% CI: 1.109-2.047, *P* = 0.009). Multicollinearity diagnostics did not indicate substantial collinearity in the multivariable models, with a maximum VIF of 1.196. Finally, receiver operating characteristic (ROC) curve analysis was performed to evaluate the discriminatory ability of baseline OGTT AUC, as illustrated in **Figure 3**. The area under the ROC curve was 0.770 (95% CI: 0.648-0.892; *P* < 0.001). In this preliminary cohort, an AUC of 17.34 was identified as a candidate, sample-dependent cut-off for pharmacotherapy use, with a sensitivity of 88.0% and a specificity of 61.5%; validation in larger prospective cohorts is required before clinical application. These findings should be interpreted cautiously and warrant further validation in larger cohorts.

**Table 4:** Exploratory regression models for the associations of OGTT AUC with overall TAR and pharmacotherapy use.

Outcome	Model	Adjustment	Effect measure	Estimate	95% CI	*P* value
Overall TAR	Model 1	Unadjusted	*B*	0.551	0.234-0.867	0.001
Overall TAR	Model 2	Maternal age, pBMI	*B*	0.555	0.239-0.871	0.001
Overall TAR	Model 3	Maternal age, pBMI, mean glucose	*B*	0.196	0.009-0.383	0.040
Pharmacotherapy use	Model 1	Unadjusted	OR	1.587	1.187-2.121	0.002
Pharmacotherapy use	Model 2	Maternal age, pBMI	OR	1.585	1.185-2.120	0.002
Pharmacotherapy use	Model 3	Maternal age, pBMI, mean glucose	OR	1.507	1.109-2.047	0.009

Values are shown for the association of OGTT AUC with each outcome. For overall TAR, estimates are unstandardized regression coefficients (B) from linear regression models. For pharmacotherapy use, estimates are odds ratios (ORs) from logistic regression models. Model 1 was unadjusted. Model 2 was adjusted for maternal age and pre-pregnancy BMI. Model 3 additionally adjusted for CGM-derived mean glucose as a sensitivity analysis. OGTT, oral glucose tolerance test; AUC, area under the curve; TAR, time above range; CGM, continuous glucose monitoring; pBMI, pre-pregnancy body mass index; CI, confidence interval.

## 4 Discussion

Pregnant women with GDM have higher CGM-measured glucose levels and more hyperglycemia compared with those who do not develop GDM (14). Since the HAPO study established a continuous association between maternal hyperglycemia and adverse pregnancy outcomes (15), maintaining early glycemic stability is crucial for mitigating adverse maternal and fetal outcomes. In our 14-day continuous monitoring, we observed that patients in the highest baseline OGTT AUC quartile (the Q4 group) exhibited elevated comprehensive glycemic metrics, specifically MAGE, SD, and overall TAR, compared to those with lower AUC levels. In line with the broader emphasis on temporal CGM profiling in pregnancy (16), our study further suggests that baseline OGTT AUC is associated with subsequent CGM-derived glycemic burden. The exploratory regression analyses showed that OGTT AUC was consistently associated with both CGM-derived TAR and pharmacotherapy use. Importantly, these associations were already evident in unadjusted and minimally adjusted models including only baseline maternal characteristics. This suggests that OGTT AUC may serve as a simple marker of subsequent glycemic burden and clinical management intensity in GDM. Notably, the sensitivity models additionally adjusted for CGM-derived mean glucose require careful interpretation. Mean glucose is closely related to TAR and may represent downstream glycemic burden after baseline OGTT-defined glucose intolerance. Therefore, we did not consider mean glucose part of the primary adjustment set. Nevertheless, the persistence of the associations after additional adjustment for mean glucose suggests that OGTT AUC may capture glycemic features beyond average glucose levels, such as the frequency, duration, or temporal clustering of hyperglycemic excursions.

The linear regression analysis confirmed a positive association between baseline AUC and the overall 14-day TAR. In agreement with recent findings by Pan et al. (17), this linear relationship shows that incremental increases in baseline OGTT AUC were associated with higher overall hyperglycemic burden. This suggests that beyond serving as a diagnostic criterion, the OGTT AUC may serve as a simple quantitative marker associated with subsequent CGM-derived glycemic instability in patients with GDM, and help identify women who warrant closer CGM-based monitoring after diagnosis. Although OGTT AUC-based stratification is not currently part of standard clinical algorithms, our findings are consistent with guideline recommendations emphasizing individualized glucose monitoring and timely treatment escalation in pregnancies complicated by GDM (18).

Analysis of the 24-hour continuous glucose profiles revealed distinct temporal glucose patterns across OGTT AUC quartiles. During waking hours (06:00-22:00), the Q4 group exhibited extensive hyperglycemia and greater glycemic variability, reflected by elevated TAR and SD. Although overall nighttime metrics showed limited statistically significant differences across quartiles, subdivision of the nighttime period revealed a more apparent early-night (22:00-02:00) pattern in the Q4 group. Specifically, women in the highest OGTT AUC quartile showed higher early-night glucose levels and greater glycemic variability during the early-night window, whereas these differences were no longer evident during the late-night period (02:00-06:00). One possible explanation is that women with higher OGTT AUC may have reduced capacity to clear postprandial glucose loads, particularly after evening meals. This interpretation is biologically plausible because impaired insulin secretion, β-cell dysfunction, and increased insulin resistance are central features of GDM (19–21), and delayed postprandial glucose clearance may extend into the early-night period (22). Nocturnal glucose patterns may also be influenced by modifiable behavioral factors, including evening meal composition, delayed eating, and bedtime snacking (12). However, these mechanisms remain speculative in the present study, as we did not directly measure insulin secretion, β-cell function, dietary composition, meal timing, bedtime snacking, physical activity, or sleep patterns. Therefore, the observed early-night pattern should be interpreted as a hypothesis-generating CGM pattern rather than evidence of a specific pathophysiological mechanism. Clinically, these findings suggest that women with higher OGTT AUC may warrant closer review of evening glucose profiles and lifestyle factors that influence nocturnal glycemia. However, they should not be interpreted as supporting a specific treatment algorithm without prospective validation.

Drug treatment and lifestyle intervention are two primary approaches to managing pregnancies complicated by diabetes (23, 24). Pharmacological intervention is indicated for patients with lifestyle intervention failing to meet glycemic targets to reduce maternal-fetal complications. Thus, identifying patients most likely to require pharmacological support at an early stage is a clinical priority. In our study, 25 (39.1%) patients required pharmacological intervention, with the vast majority of these cases concentrated in the higher AUC quartiles (Q3: n = 8; Q4: n = 11). This pattern suggests that baseline OGTT AUC may help characterize the intensity of subsequent glycemic management required under routine clinical care. Previous studies, such as that by Zhang et al., have shown that higher AUC values imply a higher risk of adverse perinatal outcomes (25). In contrast, no significant differences in maternal-neonatal outcomes were observed across quartiles; however, this finding should be interpreted cautiously because of the small sample size and low event frequency.

Technological advancements have made CGM increasingly accessible for routine clinical use. Patients exhibit a marked preference for CGM over traditional SMBG, as it facilitates more convenient glucose management without the discomfort of frequent finger-pricking (26). For patients with high OGTT AUC levels, CGM may provide additional information on nocturnal and postprandial glycemic excursions that may be missed by intermittent SMBG. This allows for the identification of hidden glycemic excursions, ensuring a more comprehensive assessment of metabolic risk in high-risk pregnancies.

Our study has several key strengths. First, the use of a 14-day CGM period provided a robust and comprehensive dataset, allowing for a more granular identification of distinct glycemic patterns across different OGTT AUC quartiles that might be missed by shorter monitoring durations. Second, this study suggests that OGTT AUC may provide additional information for risk characterization beyond conventional diagnostic thresholds. However, this study has several limitations. First, this was a single-center, retrospective study with a relatively small sample size (n = 64), which may limit the generalizability of the findings. Second, the low frequency of adverse perinatal events may have reduced the statistical power to detect differences in certain maternal and neonatal outcomes. Third, pharmacotherapy use was a real-world clinical management outcome rather than a protocol-defined endpoint, and treatment decisions may have been influenced by post-diagnostic glucose monitoring and clinician judgment. Because treatment-initiation dates were incompletely documented, the exact temporal sequence between CGM monitoring and pharmacotherapy initiation could not be reliably verified for all participants. Pharmacotherapy during CGM monitoring may have influenced CGM-derived metrics, particularly mean glucose and TAR, and may have attenuated differences in hyperglycemia exposure between quartile groups. Accordingly, associations among OGTT AUC, CGM-derived metrics, and pharmacotherapy use should be interpreted as exploratory rather than as definitive prediction rules. Fourth, CGM-derived mean glucose may lie downstream of OGTT AUC and may also contribute to clinical treatment decisions; therefore, models including mean glucose were treated as sensitivity analyses. In addition, selection bias cannot be excluded because the cohort was derived from an institutional CGM registration log and participants with sensor-related interruption of CGM monitoring were excluded. Finally, the ROC-derived AUC cut-off requires validation in larger prospective cohorts with standardized treatment-initiation criteria before clinical application.

## 5 Conclusion

In conclusion, higher OGTT AUC was associated with greater CGM-derived hyperglycemic exposure, a possible early-night glycemic pattern, and a higher likelihood of pharmacotherapy use in this GDM cohort. OGTT AUC may help support risk characterization and targeted glucose monitoring, particularly during the early-night period. Given the potential long-term implications of maternal hyperglycemia for offspring metabolic health (27), these findings warrant validation in larger prospective cohorts with standardized treatment-initiation criteria.

## Data Availability

The raw data supporting the conclusions of this article will be made available by the authors, without undue reservation.
